# Novel prism shaped C_3_N_4_-doped Fe@Co_3_O_4_ nanocomposites and their dye degradation and bactericidal potential with molecular docking study

**DOI:** 10.1039/d1ra03949k

**Published:** 2021-07-02

**Authors:** Syed Ossama Ali Ahmad, Muhammad Ikram, Muhammad Imran, Sadia Naz, Anwar Ul-Hamid, Ali Haider, Anum Shahzadi, Junaid Haider

**Affiliations:** Solar Cell Application Research Lab, Department of Physics, Government College University Lahore Lahore 54000 Punjab Pakistan dr.muhammadikram@gcu.edu.pk; State Key Laboratory of Chemical Resource Engineering, Beijing Advanced Innovation Centre for Soft Matter Science and Engineering, Beijing Engineering Center for Hierarchical Catalysts, Beijing University of Chemical Technology Beijing 100029 China; Tianjin Institute of Industrial Biotechnology, Chinese Academy of Sciences Tianjin 300308 China; Core Research Facilities, King Fahd University of Petroleum & Minerals Dhahran 31261 Saudi Arabia anwar@kfupm.edu.sa; Department of Clinical Medicine and Surgery, University of Veterinary and Animal Sciences Lahore 54000 Punjab Pakistan dralihaider99@gmail.com; Punjab University College of Pharmacy, University of the Punjab Lahore 54000 Pakistan

## Abstract

Novel prism shaped C_3_N_4_-doped Fe@Co_3_O_4_ nanocomposites were fabricated *via* a co-precipitation route for effective removal of organic pollutants from water and for bactericidal applications. Doping of C_3_N_4_ in the heterojunction significantly enhanced the photocatalytic and sonocatalytic activity against methylene blue ciprofloxacin (MBCF) dye. The main purpose of doping Fe atoms in the cobalt lattice was to generate crystal and surface defects. Moreover, the optimum doping amount of C_3_N_4_ for maximum degradation performance was evaluated. A detailed examination of the prepared nanocomposites was carried out systematically using various characterization tools for better understanding. HR-TEM images revealed the formation of novel prism shaped structures that exhibited outstanding degradation of the organic dye in water. Significant bactericidal potential was also observed for the synthesized nanocomposites against *Escherichia coli* (*E. coli*) and *Staphylococcus aureus* (*S. aureus*) bacteria. *In silico*, molecular docking studies against β-lactamase, DHFR and FabI enzymes served to elucidate the mechanism governing the bactericidal activity of the as-synthesized nanoparticles (NPs). Furthermore, a scavenging study by DPPH (2,2-diphenyl-1-picrylhydrazyl) assay and COD (chemical oxygen demand) analysis was performed in order to evaluate active species and the anti-oxidant potential of prepared composites.

## Abstract 2

This study provides new insights into the use of cobalt-based heterojunction photocatalysts for dye degradation and antibacterial applications (a) synthesis mechanism of C_3_N_4_ (b) schematic of synthesis route adopted for fabrication of nanocomposites.

## Graphical abstract

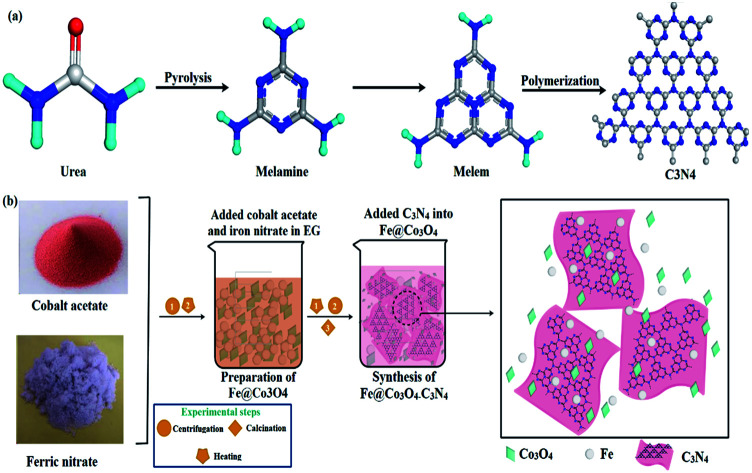

## 1 Introduction

Water is the prime necessity for all life present on this planet. Water pollution, being a serious threat to life on Earth, poses a grave challenge for humanity to overcome.^1–4^ About 1 billion people are affected every year by water pollution and about 1.8 million deaths were recorded in the year 2015 according to a report published in *The Lancet*. A major source of water pollution are the organic dyes emanating from industrial wastes which are extremely harmful due to their carcinogenic nature.^5,6^ Various techniques developed by researchers in the past for water treatment are costly and energy-consuming.^7–12^ A recent and cost-effective method that has emerged is the use of photocatalysts for water treatment and water splitting.^13–19^ Apart from its numerous advantages over other techniques, a major drawback while using semiconductor photocatalysts is the corrosion and dissolution of catalysts in aqueous medium when irradiated with light. High recombination rate and agglomeration also influence their performance as catalysts. Researchers are looking for catalysts that are more stable under light and have low recombination and agglomeration rates, which will serve to improve performance.^13^

Heterojunction photocatalysts are produced to address the drawback of high recombination rates of semiconductors.^20–24^ Heterojunctions, however, in bulk form are not effective and use of nanomaterials becomes necessary to boost their performance. Large surface area and high reactivity of nanocatalysts play an important role in photocatalysis for the removal of organic pollutants from water.^25^ Among 2D nanomaterials, graphitic carbon nitride (g-C_3_N_3_) has emerged as a strong candidate for photocatalysis, possessing mid-range band gap (2.7 eV) and tunable electronic properties.^26–28^ However, g-C_3_N_3_ suffers from high recombination rate, poor visible light absorption and small surface area. Various nanostructure designs have been synthesized to increase the performance of g-C_3_N_3_ photocatalysts.^29–31^ Production of g-C_3_N_3_ based heterojunctions increases catalysts' charge transfer efficiency to improve the overall photocatalytic performance.^32,33^ Moreover, addition of co-catalysts into the framework has shown to yield outstanding results by enhancing stability and activity of g-C_3_N_3_ based catalysts.^34^

Plenty of work has been reported recently on g-C_3_N_3_ based photocatalysts embedded with various metal oxides such as ZnO, TiO_2_ and WO_3_ that are used as co-catalysts.^34–36^ Among such co-catalysts, Co_3_O_4_ emerged as a viable candidate for photocatalytic activity. Co_3_O_4_ grasped the attention of researchers after the work of Liao *et al.* on the degradation of water using Co_3_O_4_ under visible light irradiation.^37^ Vivid optical response under visible light and good charge transfer efficiency of Co_3_O_4_ (*B*_g_ = 2.6 eV) makes it highly valuable.^28,38^ One difficulty, however, is the severe aggregation of Co_3_O_4_ nanoparticles, which causes massive reduction in photocatalytic activity.^39^ To overcome this, suitable semiconductors can be doped in Co_3_O_4_ or highly refined synthesis techniques could be adopted to fabricate dispersed Co_3_O_4_ nanoparticles.^40^ Appropriate solvents can significantly affect the size, morphology and aggregation behavior of nanomaterials. In this regard, ethylene glycol (EG) has been reported as an effective solvent for suppressing severe aggregation of nanoparticles. Moreover, it can act as a surfactant also to regulate the uniformity of NPs diameter.^41^ No work has been reported yet (to best of our knowledge) on the use of C_3_N_4_-doped Co_3_O_4_ composites for dye degradation and anti-bacterial applications.

**Fig. 1:**
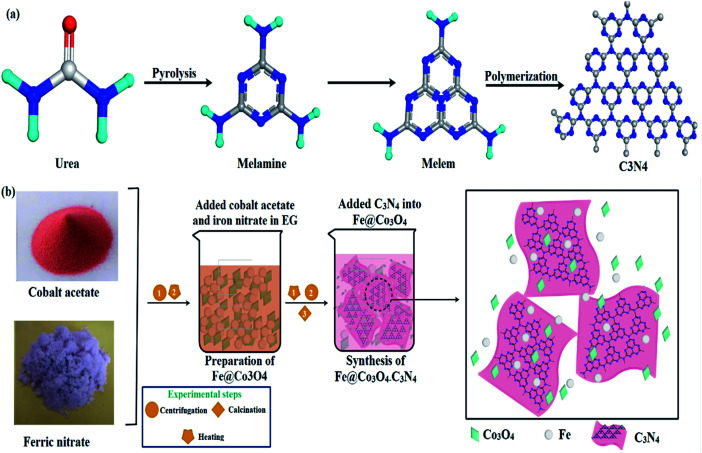
(a) Synthesis mechanism of C_3_N_4_. (b) Schematic representation of synthesis route adopted for fabrication of nanocomposites.

In the following work, a bottoms-up technique (co-precipitation) has been adopted to synthesize C_3_N_4_-doped Fe@Co_3_O_4_ nanostructures for photocatalytic degradation of methylene blue ciprofloxacin (MBCF) dye. A schematic representation of the synthetic route adopted is given in Fig. 1. Moreover, bactericidal activity of as-prepared samples was evaluated against Methicillin Resistant (MR) *E. coli* and *S. aureus* bacteria and possible mechanism for anti-microbial activity has been proposed using molecular docking studies. The docking predictions were performed against β-lactamase, DHFR and FabI enzymes belonging to cell wall, folate and fatty acid biosynthetic pathways. Structural and optical properties, chemical composition and surface morphology of various doped samples were studied. Moreover, comparison of pure Co_3_O_4_ particles and doped Co_3_O_4_ has also been provided for better understanding.

## 2 Experimental section

### 2.1. Materials

Ethylene glycol (EG) and cobalt acetate (Co(CH_3_CO_3_O_4_)_2_·4H_2_O) were purchased from MERCK and PANREAC, respectively. Iron nitrate (Fe(NO_3_)_3_·9H_2_O) was received from UNICHEM and NaOH from SIGMA ALDRICH. Carbon nitride (C_3_N_4_) was produced in the lab *via* pyrolysis of urea (CH_4_N_2_O).

### 2.2. Synthesis

5 g of Co(CH_3_CO_3_O_4_)_2_·4H_2_O and 0.25 g of Fe(NO_3_)_3_·9H_2_O were added in EG (60 mL) under vigorous stirring at 75 °C to form a homogeneous mixture. Various amounts of C_3_N_4_ (50, 100, 150 mg) were added in the mixture and sonicated for 4 hours. NaOH solution was used to maintain pH of the samples. Sonicated mixtures were centrifuged and annealed at 400 °C for 4 hours to prepare nanomaterials. The prepared samples were denoted as Co_3_O_4_, Fe@Co_3_O_4_ and C_3_N_4_ (1, 2, and 3%) where 1, 2 and 3% corresponded to various amounts of C_3_N_4_ doped in Fe@Co_3_O_4_ (Fig. 1).

### 2.3. Isolation and identification of *S. aureus* and *E. coli*

Sheep blood agar (5%) was utilized to culture mastitic milk samples (Bovine) collected from various veterinary (private and public) hospitals in Punjab, Pakistan. After incubating (for 24 hours) at 37 °C, samples were further purified by streaking them in triplicate on Manitol Salt Agar (MSA) and MacConkey agar (MA) media to isolate *S. aureus* and *E. coli* effectively. Morphological study (Gram staining) and biochemical tests (Catalase and Coagulase) were employed for the identification of isolated colonies.

### 2.4. Antimicrobial activity

Agar well diffusion phenomenon was adopted to analyze the antimicrobial activity of as-obtained samples on isolated Gram positive and negative bacteria. After swabbing MSA-deposited Petri dishes with isolated *S. aureus* and *E. coli* cultures (1.5 × 10^8^ CFU mL^−1^), wells of diameter 6 mm were generated in the dishes *via* sterilized cork borer. Various constitutions ∼0.5 mg/50 μL and 1 mg/50 μL corresponding to low and high concentration respectively, of each sample were poured into the wells along with positive control (ciprofloxacin 0.005 mg/50 μL) and negative control (DIW 50 μL). Further incubation of prepared dishes was conducted at 37 °C for 24–48 hours to investigate the antibacterial activity of nanostructures. Inhibition zones formed after 48 hours of incubation were measured using Vernier caliper (mm) and their relation with doping concentration was analyzed. One-way analysis of variance (ANOVA) was employed in order to calculate antibacterial efficacy of nanostructures in terms of inhibition zone diameter (mm).

### 2.5. Scavenging (DPPH assay)

The traditional DPPH scavenging assay was adopted with some modifications in order to check the free radical active species and anti-oxidant behavior of the prepared nanostructures. Bare and doped Co_3_O_4_ nanoparticles were utilized with various concentrations (0–500 μg mL^−1^) along with equal volume of 1 mM DPPH solution for evaluation. The reaction mixture was vortexed and incubated for 30 minutes in dark at ambient temperature. For reference sample, standard solution of ascorbic acid was used as strong anti-oxidant. The % scavenging activity of each sample was evaluated by measuring the degradation in maximum absorbance wavelength of DPPH solution (*λ* = 517 nm) as given in eqn (1).1
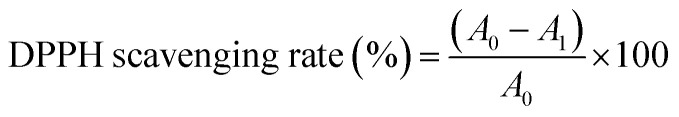
where, *A*_0_ is absorption of control (methanol + DPPH) and *A*_1_ corresponds to the absorbance value in the presence of sample after some time.

### 2.6. COD analysis

Chemical oxygen demand (COD) analysis is another important factor that decides the amount of oxygen required for complete oxidation of organic pollutant in wastewater. The conventional ferrous ammonium sulfate (FAS) assay was adopted in order to evaluate the COD of dye solution before and after degradation under visible light irradiation. The prepared sample solutions were refluxed with standard solution of potassium dichromate (K_2_Cr_2_O_7_) and sulfuric acid (H_2_SO_4_) and excess of K_2_Cr_2_O_7_ was titrated against FAS to check the COD values. Total amount of K_2_Cr_2_O_7_ consumed in the procedure offers the extent of oxygen consumed by the solution for complete oxidation of organic pollutants. The following relation was used to calculate the COD values at various irradiation time (eqn (2));2
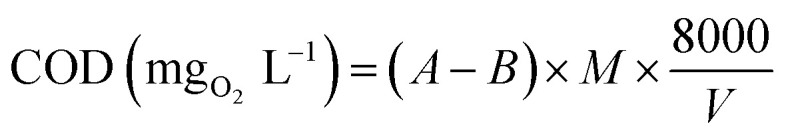
where, *A* = volume of FAS used for blank (mL), *B* = volume of FAS used for sample (mL), *M* = molarity of FAS, *V* = volume of sample.

### 2.7. Molecular docking studies

The promising antibacterial potential of C_3_N_4_-doped Fe@Co_3_O_4_ NPs revealed by *in vitro* studies prompted us to perform *in silico* molecular docking studies to identify possible interactions of NPs against selected enzyme targets. Here, we performed molecular docking predictions of NPs against key enzymes of cell wall biosynthetic pathway, folate biosynthesis and fatty acid biosynthetic pathway *i.e.* β-lactamase, dihydrofolate reductase (DHFR) and enoyl-[acyl-carrier-protein] reductase (FabI), respectively.

The 3D-structural coordinates of β-lactamase, DHFR and FabI for *S. aureus* were retrieved from protein data bank having ID as 1MWU (resolution: 2.60 Å),^42^2W9H (resolution: 1.48 Å)^43^ and 6TBC (resolution: 2.55 Å),^44^ respectively. The docking predictions were performed using molecular operating environment (MOE) software^45^ according to the method reported in our previous studies.^46,47^ The main steps involved were removal of water and native ligand followed by addition of H-atoms and ultimately energy minimization of retrieved enzyme structures. Default parameters of energy minimization algorithm (MMFF94x force field, gradient: 0.05) of MOE were utilized for energy minimization. Later, systematic conformational search was employed on these selected enzymes using default parameters of Site finder tool *i.e.* RMS gradient of 0.001 kcal mol^−1^ and binding pocket was specified within close vicinity (*i.e.* 10 Å) of native ligand. Finally, 10 top scored docking conformations were generated in each case and the best-performing complexes were further evaluated for binding tendency prediction of NPs. Pymol software was employed for analysis of interactions and 3D view representation of docked complexes. Ligand structures were prepared using builder tool of MOE software.

### 2.8. Materials characterization

X-ray powder diffraction (XRD) measurements were carried out by collecting data from 5° to 80° (2*θ* range) using a PANalytical-Xpert-PRO diffractometer with Cu-Kα radiation of *λ* = 1.5418 Å to ascertain the crystallite size and phase constitution of nanomaterials. FTIR (Fourier Transform Infrared) spectra with PerkinElmer spectroscopy was employed to detect the presence of functional groups in undoped and co-doped CuO samples while Raman spectra were obtained through a Raman Thermoscientific microscope equipped with 532 nm laser (6 mW). To observe the optical properties, UV-vis (ultraviolet-visible) and photoluminescence (PL) pictures of the samples were recorded *via* UV-vis (Genesys10S spectrophotometer) and PL analyzer (JASCO, FP-8300), respectively. Using INCA EDS software, elemental composition was obtained through energy dispersive X-ray spectroscopy. Inter-planner *d*-spacing of the synthesized products were measured using HR-TEM equipment JEOL JEM 2100F.

## 3 Results and discussion

**Fig. 2:**
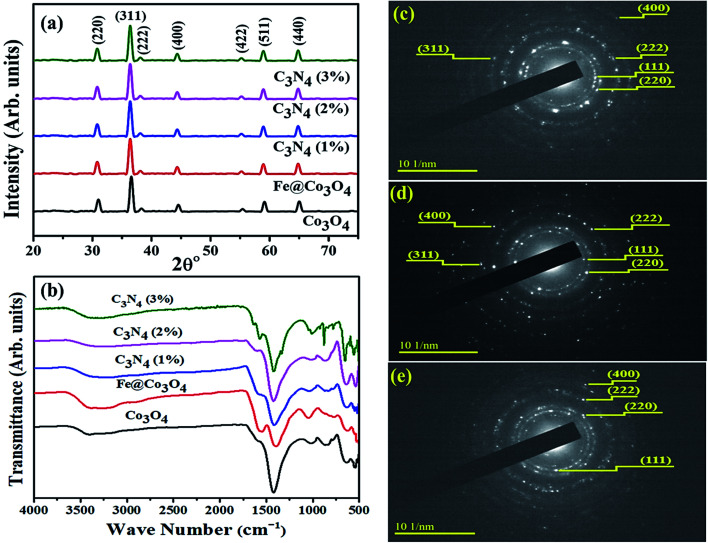
(a) XRD patterns of prepared samples, (b) Raman spectra of prepared samples and SAED patterns for (c) Co_3_O_4_, (d) C_3_N_4_ (1%) and (e) C_3_N_4_ (2%).

Specimens were characterized for structural and phase composition analysis with XRD (Fig. 2a). Diffraction peaks recorded at 2*θ*° values 31.17°, 36.65°, 38.35°, 44.35°, 55.17°, 58.98° and 65.11° consistent with crystallographic planes (220), (311), (222), (400), (422), (511) and (440) respectively, revealed the FCC cubic crystal structure (*Fd*3̄*m*) of Co_3_O_4_ (JCPDS card # 80-1534). Addition of Fe into Co_3_O_4_ caused peaks to shift slightly toward lower *θ* values, which may be attributed to a small change in lattice constant of the cobalt crystal.^48^ This slight change in lattice parameters of Fe@Co_3_O_4_ could be assigned to the difference in the atomic radii of Co (0.125 nm) and Fe (0.126 nm) or some intrinsic crystal defects.^49^ However, this confirms successful incorporation of Fe into crystal lattice.^50,51^ No significant change in peaks position and intensity was observed upon C_3_N_4_ doping into the composite. This indicates that the two-dimensional C_3_N_4_ was not incorporated in the cobalt lattice and caused no significant structural changes. It may have covered the Fe@Co_3_O_4_ nanoparticles or served as host matrix for particle decoration/incorporation. Furthermore, no characteristic peak of C_3_N_4_ appeared in any of the doped samples' spectra which is attributable to its relatively low content in the matrix.^52^ Chemical composition and functional group identification of samples was investigated *via* non-destructive FTIR analysis (Fig. 2b). Spectra were observed at room temperature in the frequency range of 500–4000 cm^−1^ and two noteworthy peaks were observed at 546 cm^−1^ and 645 cm^−1^ for Co_3_O_4_ nanostructures. The peaks attributed to 546 cm^−1^ and 645 cm^−1^ depict the stretching of Co–O bond and bridging vibration of O–Co–O bond, respectively.^53–55^ Broad bands near 3412 cm^−1^ and 1420 cm^−1^ refer to the stretching and bending vibrations of –OH group respectively, which originate from the moist environment in which samples were prepared. Bands appearing near 1428 cm^−1^ are ascribed to CO_3_ vibration in carbonate anions that might have originated due to the precursors used during synthesis (Co(CH_3_CO_3_O_4_)_2_·4H_2_O).^56–58^

Selected area electron diffraction (SAED) patterns for Co_3_O_4_ and C_3_N_4_ (50 mg, 100 mg) are shown in Fig. 2c–e. Images with concentric rings indexed with (111), (220), (222), (311) and (400) planes, are in agreement with XRD results thus confirming the FCC cubic crystal structure of Co_3_O_4_ nanostructures. Interlayer spacing for Co_3_O_4_ (*d* = 0.247) was calculated from most intense peak (311) and verified by TEM observation using Digital Micrograph (Fig. 4).

**Fig. 3:**
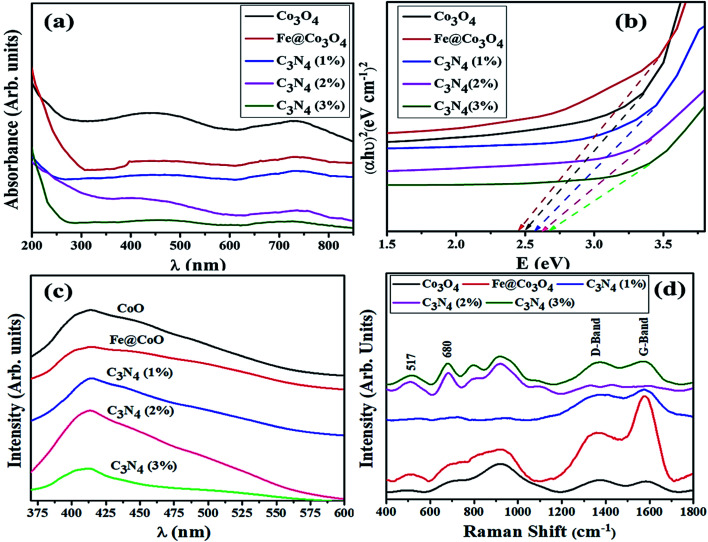
(a) UV-Vis spectra, (b) Tauc plot, (c) PL spectra and (d) Raman spectra of pristine and doped samples.

Optical absorption spectra was investigated *via* UV-Vis spectrometer at room temperature using dispersed solutions of nanocatalysts in water (Fig. 3a). Two distinct absorption windows were observed for Co_3_O_4_ around 430 nm and 750 nm that corresponded to charge transfer between metal and ligands. First absorption peak (*λ* < 500 nm) is ascribed to O_2_^−^ to Co^2+^ charge transfer while second absorption window (*λ* > 700 nm) refers to O_2_^−^ to Co^3+^ charge transfer.^59,60^ Such charge transfer is ascribed to d (t_2g_) to d (t_2_) electronic transition from t_2g_ (Co^3+^) to *t*_2_ (Co^2+^) states and p to d transition involving p (O^2−^) states of valence bands, respectively.^61^ After coupling with Fe, redshift was observed and similar behavior was noted upon doping C_3_N_4_ in the composite. This implies that C_3_N_4_ can be incorporated into doped metal oxide frameworks to enhance photo absorptivity in the visible region. The inferred absorption data has also been used to calculate band gap energies of specimens using the classical Tauc transformation for near edge absorption. After extrapolating linear portion of (αh*ν*)^2^*vs. hν* plot on *x*-axis (Fig. 3b), allowed direct band gap for Co_3_O_4_ was calculated to be 2.5 eV which is in agreement with reported literature.^62,63^ Trivial change in band gap energies for Fe@Co_3_O_4_ was observed for samples with increasing amount of C_3_N_4_ dopant concentration ∼2.6 eV for C_3_N_4_ (3%).

In order to study the change in electron transfer efficiency and recombination rate, PL spectra for all samples were examined (Fig. 3c). Co_3_O_4_ nanostructures showed characteristic PL band from 410 to 425 nm (*λ*_exc_ = 280 nm) with noteworthy decrease in peak intensity upon introduction of Fe. A substantial drop in peak intensity, which corresponds to low recombination rate and high electron transfer efficiency, was detected for low amount of doped-C_3_N_4_ in the composite as well. Reduction in intensity might be due to the defects and trap sites introduced into the structure upon doping with C_3_N_4_. This can be understood by SRH (Defect Assisted) recombination process explained by Shockley, Read and Hall in 1952.^64^ Generation of new energy levels in the forbidden band gap of Fe@Co_3_O_4_ after doping with C_3_N_4_ act as trap sites for electrons and holes, hence reducing recombination rate. Further increase in doping concentration (C_3_N_4_ 2, 3%) caused an increase in PL intensity indicating reduction in electron transfer efficiency. This may be attributed to substantial agglomeration of particles or formation of 2d sheets of C_3_N_4_ over the surface of nanostructures.

Room temperature Raman spectroscopy was utilized to further investigate phase composition and structural defects present in prepared nanostructures (Fig. 3d). Characteristic bands observed at 517 cm^−1^ and 680 cm^−1^ were attributed to F^2^_2g_ and A_1g_ Raman-active modes of Co_3_O_4_ nanoparticles. This confirmed the cubic spinel structure of Co_3_O_4_ belonging to *Fd*3̄*m* group with Co^3+^ and Co^2+^ atoms at tetrahedral and octahedral sites, respectively.^65^ Two bands near 1365 cm^−1^ and 1580 cm^−1^ are respectively referred to as D-band (defective band) and G-band of carbon containing materials. The D-band (sp^2^ Raman signature) which arises due to TO (transverse optical) phonons near k-point indicates the defects present in carbon hexagons breathing modes. On the other hand, G-band is the consequence of E_2g_ phonons at the center of Brillouin zone, indicating in-plane C–C stretching (double-degenerated) mode which is characteristic Sp^2^ Raman signature of carbon based materials.^66,67^ The ratio of G and D bands intensity (*I*_D_/*I*_G_) can be used to evaluate the extent of disorder present in carbonaceous materials. Minimum *I*_D_/*I*_G_ ratio (0.99) was calculated for C_3_N_3_ (3%) confirming the least amount of defects.

**Fig. 4:**
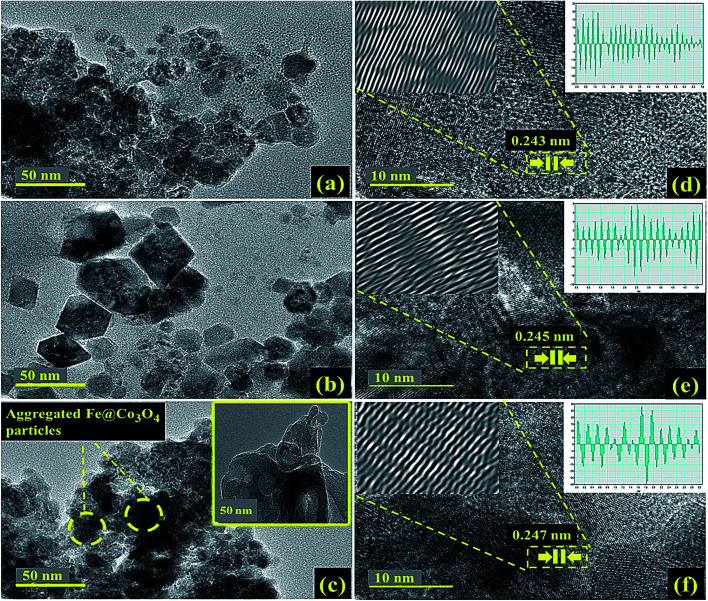
HR-TEM images of (a) Co_3_O_4_, (b) C_3_N_4_ (1%), (c) C_3_N_4_ (2%) with inset of pristine C_3_N_4_ and (d–f) *d*-spacing evaluation of corresponding samples.

Morphological and structural study of pure and doped samples was carried out using HR-TEM images up to 10 nm resolution (Fig. 4). As expected, pure Co_3_O_4_ depicted aggregated structure with particles of size less than 15 nm (Fig. 4a). Prism shaped particles of size 25–35 nm were observed upon 1% doping with C_3_N_4_ as shown in Fig. 4b. These prism-like structures exhibit improved catalytic performance by offering large surface area and increased number of active sites. For higher dopant concentration of C_3_N_4_, TEM images (Fig. 4c) revealed aggregated Fe@Co_3_O_4_ nanoparticles uniformly decorated on C_3_N_4_ sheets, hence confirming the presence of heterojunction between C_3_N_4_ and Fe@Co_3_O_4_.^28^ Highly magnified images (10 nm) were used to show lattice fringes for identification of crystallographic planes to calculate *d*-spacing. The *d*-spacing calculated for Co_3_O_4_ came out to be ∼0.243 nm that is consistent with theoretical *d*-spacing of (111) crystallographic plane of Co_3_O_4_, Fig. 4d–f.

**Fig. 5:**
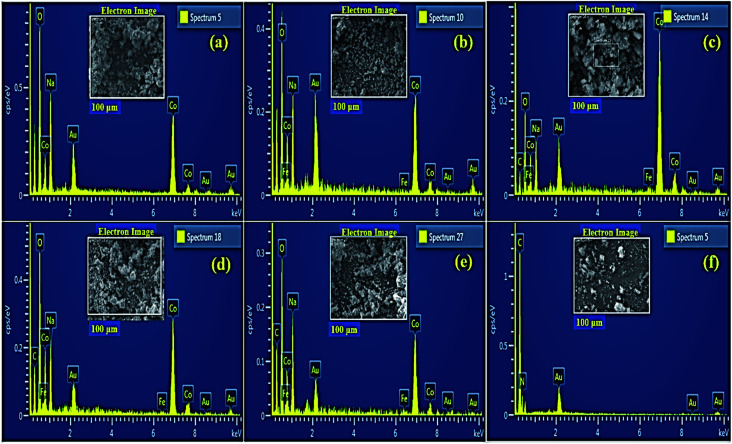
EDS profiles of (a) Co_3_O_4_ (b) Fe@Co_3_O_4_ (c) C_3_N_4_ (1%) (d) C_3_N_4_ (2%) (e) C_3_N_4_ (3%) and (f) pristine C_3_N_4_.

Electron dispersive X-ray spectroscopy (EDS) was carried out for elemental composition tracing of obtained nanostructures as depicted in Fig. 5a–f. Strong peaks of Co, Fe and O confirmed the presence of Fe@Co_3_O_4_ composite while additional peaks of C and N assured the successful incorporation of C_3_N_4_ into crystal lattice. Small amount of Na was detected which might have originated due to the use of NaOH to maintain pH of samples during synthesis.

The magnetic properties of bare and doped Co_3_O_4_ materials have been previously reported in the literature. In a typical Co_3_O_4_ spinel structure, the magnetic moment mainly arises due to Co^2+^ species while Co^3+^ ions do not exhibit any permanent magnetic moment. This is mainly owed to the fully filled t_2g_ orbitals arising from splitting of 3d orbitals in octahedral crystal field.^68^ Furthermore, the bulk state Co_3_O_4_ structures exhibit antiferromagnetic behavior, but the nanostructures of cobalt have been reported to possess a slight ferromagnetic nature due to quantum size effects and uncompensated spins.^69^ In addition to that, Co_3_O_4_ nanorods demonstrate higher coercivity values in contrast to nanoparticles as well, which is owed to the shape anisotropy.^70^ Studies have also revealed that doping of Fe also induces a slight ferromagnetism in Co_3_O_4_ nanostructures.^71^

### 3.1. Photocatalytic activity

**Fig. 6:**
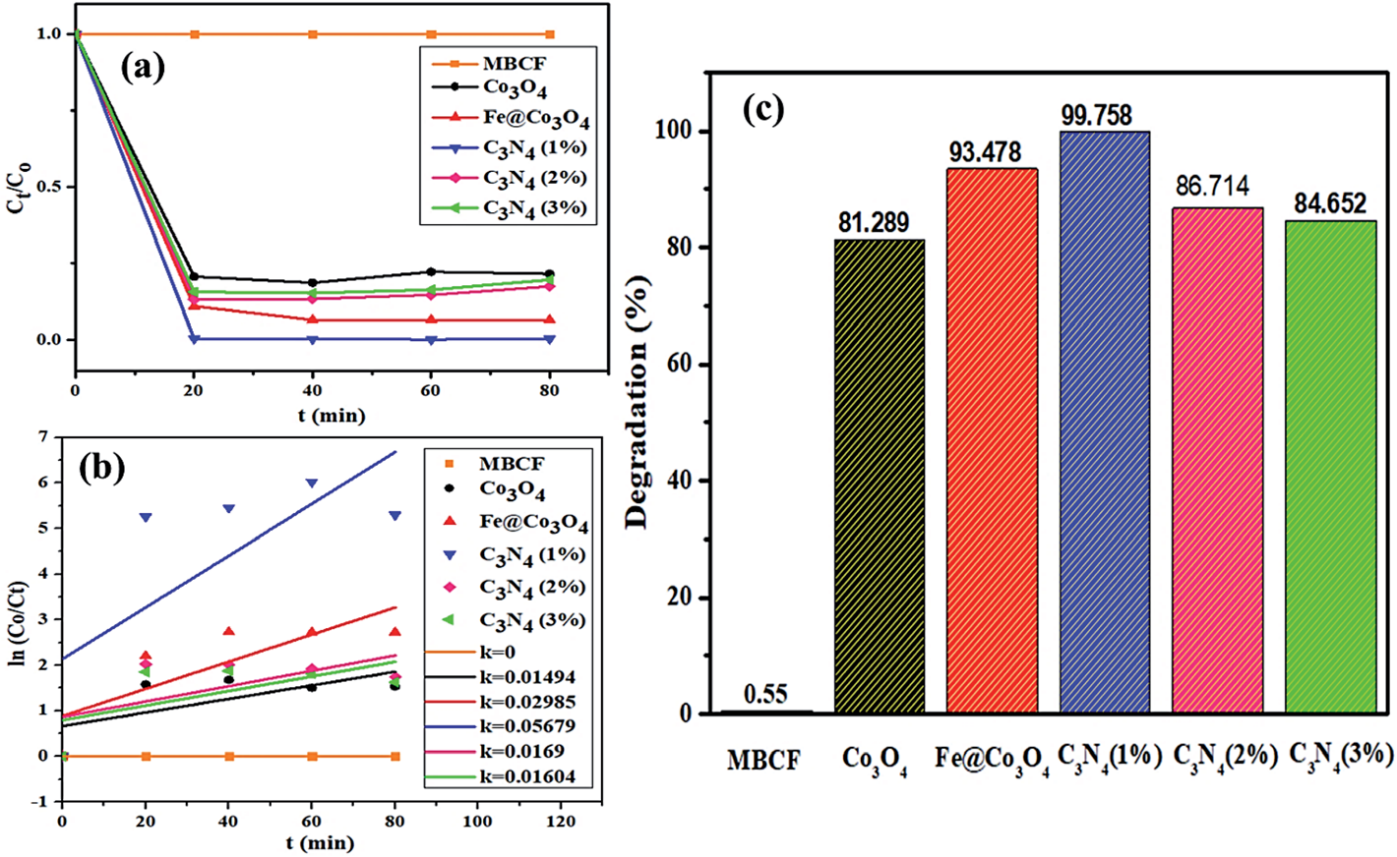
Photocatalytic activity of prepared nanocatalysts (a) plot of concentration ratio (*C*_*t*_/*C*_o_) *vs.* time, (b) plot of ln(*C*_o_/*C*_*t*_) *vs.* time, (c) plot of % degradation performance.

Photocatalytic activity of as-obtained samples was measured under visible light irradiation (Fig. 6). Time dependency of absorbance was plotted for solutions containing 10 mg of samples in 40 mL of MBCF. Furthermore, the pH of the due solution was measured to be 6.1 and the evaluations were conducted in this slightly acidic pH. The solution was placed under visible light irradiation and 3 mL of dye was syringed out at regular intervals of time for UV-Vis analysis. Mercury lamp with 400 W intensity and wavelength ranging from 400–700 nm was employed as the irradiation source. Reduction in absorption maxima (*λ*_max_ = 665 nm) was observed which evidenced the reduction of dye significantly. Maximum degradation of dye was observed just within 40 minutes for all nanocatalysts. Co_3_O_4_ showed 81% degradation of dye after 40 minutes of light irradiation and upon Fe doping, degradation performance significantly increased to 93% for the same duration. Upon 1% doping of C_3_N_4_ in Fe@Co_3_O_4_, remarkable increase in photocatalytic activity was observed with maximum degradation of 99% noted within 40–45 minutes. Decrease in dye degradation performance was observed for higher doping concentration of C_3_N_4_ (2 and 3%) implying that the optimum doping concentration of C_3_N_4_ in Fe@Co_3_O_4_ was 1% for best photocatalytic performance. Moreover, rate constants were calculated by applying pseudo first order kinetics to linearly fitted curves of ln(*C*_o_/*C*_*t*_) *vs.* time plot from eqn (3) as follows:3
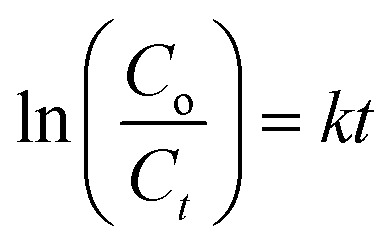


Increase in photocatalytic performance of Co_3_O_4_ upon doping of Fe and C_3_N_4_ can be attributed to reduced recombination rate of photo-induced charge carriers as depicted by PL spectroscopy (Fig. 3c). Apart from that, degradation performance majorly depends upon size, shape and surface area of nanocatalysts. Particles with large surface area provide greater number of active sites for atoms or molecules hence increasing the number of redox reactions, leading to degradation of MB to LMB. The photo-generated electrons and holes interact with surrounding oxygen (O_2_) and water (H_2_O) molecules to form highly reactive ˙O_2_^−^, ˙OH^−^ and H^+^ radical species, respectively. The excessive H^+^ ions and electrons combine with dye molecules leading to the reduction of MB to LMB.^72^

Addition of Fe atoms into Co_3_O_4_ boasts the electron transfer efficiency of nanocatalysts by introducing defects in the lattice. This defect-assisted recombination phenomenon has been previously discussed in PL spectra analysis where defects introduced in the lattice act as trap sites for electrons and holes rendering their recombination.^64^ Doping of C_3_N_4_ into Fe@Co_3_O_4_ also compliment the above-mentioned phenomenon but only to a certain extent of doping concentration. Further increase in doping amount reduces the catalytic activity of nanostructures which may be attributed to agglomeration of particles or the predominance of 2D sheet-like structure of C_3_N_4_ enclosing the particles within it.

**Fig. 7:**
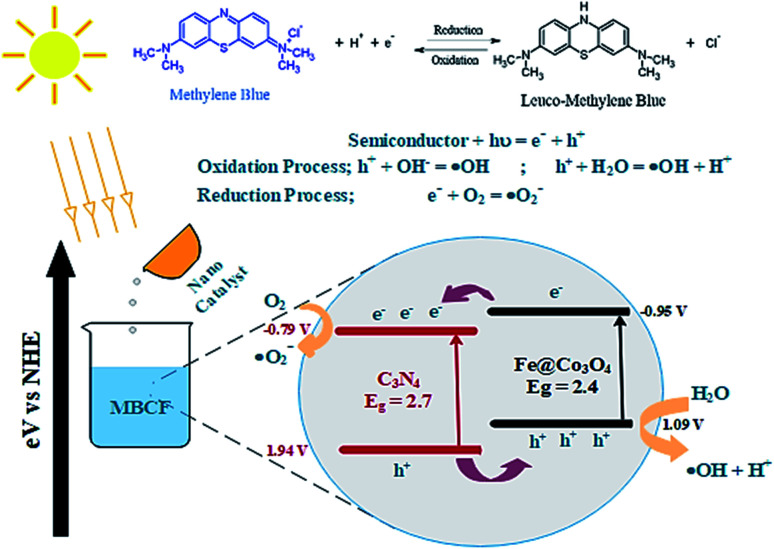
Schematic illustration of proposed mechanism involved in the photocatalytic degradation of MBCF dye over C_3_N_4_/Fe@Co_3_O_4_ catalyst.

A possible mechanism for the photocatalytic degradation of MB over the prepared nanocatalysts is proposed in Fig. 7. The Fe@Co_3_O_4_ exhibits a lower VB (valence band) and a higher CB (conduction band) values in contrast to C_3_N_4_ sheets which significantly result in better separation of photogenic charge carriers. As a consequence, electrons from CB of Fe@Co_3_O_4_ composite jump to the CB of C_3_N_4_ while the holes in the VB of C_3_N_4_ get transferred to Fe@Co_3_O_4_ atoms. This favors a type-II carrier transfer in the heterojunction resulting in efficient separation of photogenic excitons. The E_CB_ of doped C_3_N_4_ is more negative in contrast to *E*_0_ of O_2_/˙O_2_^−^ (−0.33 eV). Hence, the electrons in CB of C_3_N_4_ significantly reduce the O_2_ species into reactive radical ˙O_2_^−^ species whereas the holes in the VB interact with surrounding H_2_O molecules and oxidize them to form radical ˙OH^−^ species. These radical species interact with the surrounding dye molecules and complete degradation of organic pollutant occurs. The MB dye being cationic in nature get reduced by accepting electrons from these highly reactive species. Consequently, π-conjugation occurs after addition of electrons which results in the formation of Leuco-MB which is colorless in nature.

### 3.2. Sonocatalytic activity

**Fig. 8:**
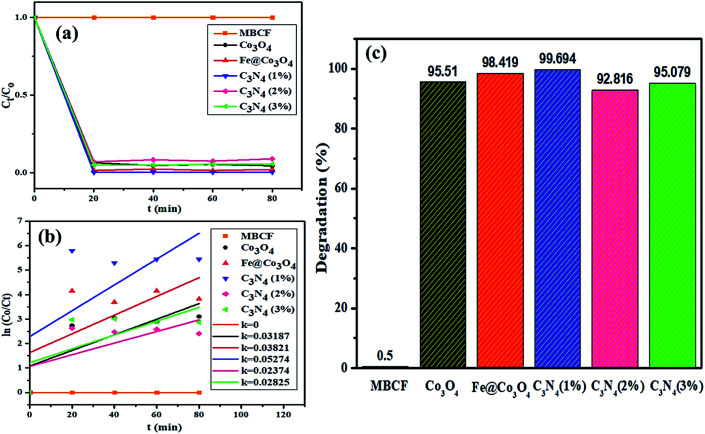
Sonocatalytic activity of nanocatalysts (a) plot of concentration ratio (*C*_*t*_/*C*_o_) *vs.* time, (b) plot of ln(*C*_o_/*C*_*t*_) *vs.* time, (c) plot of % degradation performance.

Sonocatalytic activity of nanocatalysts was also observed by sonicating the sample solution (10 mg of nanocatalyst in 40 mL of MBCF) in ultrasonic bath for at least 80 minutes in the absence of light (Fig. 8). Similarly, 3 mL of sample were taken for UV-Vis analysis at regular interval of time and degradation of dye was evaluated. Overall, sonocatalytic performance was found to be markedly better than photocatalytic activity for all samples. The maximum degradation for Co_3_O_4_ were measured to be 95% while Fe@Co_3_O_4_ showed a degradation of 98% after 40–50 minutes of sonication. Maximum degradation of 99% was observed for C_3_N_4_ (1%) whereas, decrease in degradation performance was detected for samples with higher concentration of C_3_N_4_ (2, 3%).

The mechanism involved in degradation of dye in the above-mentioned procedure can be explained *via* hot spot theory which deals with the formation of microbubbles, their nucleation, growth and collapse to produce heat and light (sonoluminescence).^73^ Ultrasonication helps in catalytic activity of nanocatalysts by following acoustic cavitation mechanism where large number of hot spots are produced after extensive energy accumulation.^74^ The compressing and expanding ultrasonic waves cause change in bubble size (*i.e.*, grow, compress) resulting in implosive collapse under extreme conditions. This implosion releases adequate amount of energy (heat and light) to generate electron hole pairs on the surface of nanocatalysts. These electrons and holes take part in redox reactions where electrons act as strong reducing agents while holes behave as strong oxidizing agent. Another consequence of this released energy is the breaking of surrounding water molecules to produce highly reactive radical H^+^, OH^−^ and O^−^ species. Addition of nanocatalyst in solution complements the formation of microbubbles while the number of active sites is significantly increased.^75^

### 3.3. Photo-sono catalytic activity

**Fig. 9:**
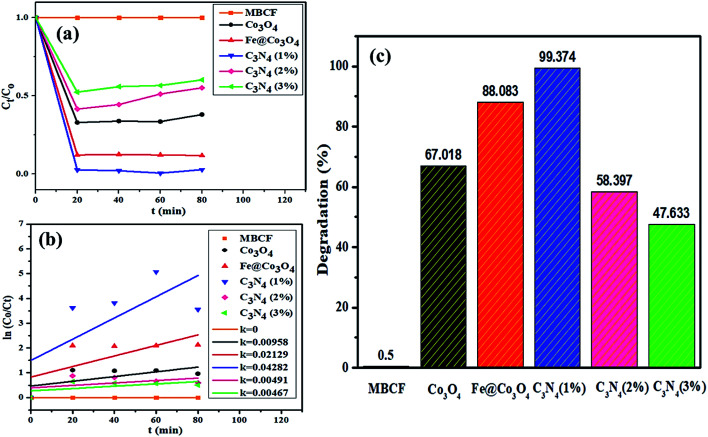
Sono-photocatalytic activity of nanocatalysts (a) plot of concentration ratio (*C*_*t*_/*C*_o_) *vs.* time, (b) plot of ln(*C*_o_/*C*_*t*_) *vs.* time, (c) plot of % degradation performance.

In order to check the combined effect of sonocatalysis and photocatalysis, samples were placed in sonicator under visible light irradiation for at least 80 minutes (Fig. 9). Surprisingly, reduction in catalytic activity was observed for almost all samples in photo-sono catalysis with Co_3_O_4_ showing maximum degradation of 67% in 80 minutes. Decrease in activity was observed for Fe@ Co_3_O_4_ as well with maximum degradation of 88% while C_3_N_4_ (1%) gave a consistent degradation of 99% within 60 minutes. Samples with higher doping concentration, C_3_N_4_ (2%) and C_3_N_4_ (3%) showed degradation up to 58% and 47%, respectively.

In addition to above, COD analysis was performed to further evaluate the best-performing photocatalyst C_3_N_4_ (1%) in dye solution under visible light irradiation in order to check the oxygen demand before and after degradation of the dye (Table 1). For evaluation, 10 mg of photocatalyst in 1000 mL of dye solution was placed under visible light for 80 minutes and 1 mL was syringed out at regular intervals to check the COD values. The blank sample with no photocatalyst showed a high COD value of 1865 mg L^−1^ which gradually decreased after light exposure in the presence of catalyst. Maximum COD degradation of up to 95% was achieved after 60 minutes of dye degradation over C_3_N_4_ (1%) catalyst. These results show that the prepared photocatalysts can act as potential candidates for pre-treatment of polluted water for effective oxidation of organic pollutants.

**Table 1:** COD degradation of MBCF dye under irradiation using C_3_N_4_ (1%) photocatalyst

Time (*t*)	COD (mg L^−1^)	COD degradation
0 minutes	1865	0%
20 minutes	1213	35%
40 minutes	560	70%
60 minutes	94	95%

### 3.4. Scavenging (DPPH) assay

**Fig. 10:**
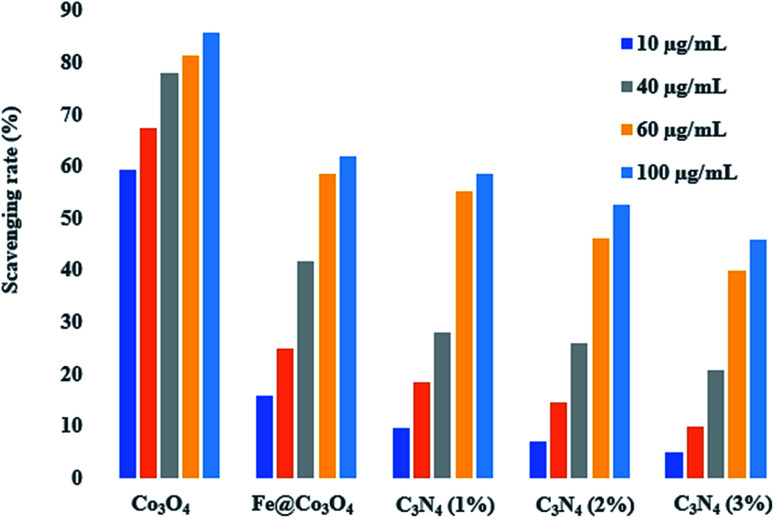
DPPH scavenging activity of bare and doped Co_3_O_4_ nanostructures.

In order to evaluate the active radical species present in the photocatalyst and to measure their anti-oxidant activities, DPPH scavenging assay was employed. Antioxidant characteristics of compounds is tied to their electron or hydrogen atom donating capability to DPPH free radical, such that they create stable diamagnetic compounds. This DPPH free radical's reduction capability can be examined spectrophotometrically by evaluating the degradation in absorbance at 517 nm. A dose dependent behavior was observed for the anti-oxidant activities of all prepared samples (Fig. 10). The maximum scavenging performance of 85.56% was exhibited by pristine Co_3_O_4_ after 120 minutes at concentration of 100 μg mL^−1^. Generation of highly reactive ˙OH and ˙O_2_^−^ radical species, which have the potential to bond with the DPPH free radical can result in its degradation. Upon Fe addition, a significant drop in anti-oxidant activity up to 61.82% was observed at the same concentration. Further decrease in scavenging performance was observed upon increasing the amount of C_3_N_4_ in Fe@Co_3_O_4_ lattice. For C_3_N_4_ (1%), maximum performance of 58.43% was evaluated while C_3_N_4_ (2%) and C_3_N_4_ (3%) samples exhibited anti-oxidant potential of 52.71% and 45.86%, respectively. This decrease in anti-oxidant potential of photocatalyst upon C_3_N_4_ addition might be attributed to the increase in turbidity of test sample, which in turn caused an antagonistic interaction, resulting in a depleted scavenging activity.^76^

**Fig. 11:**
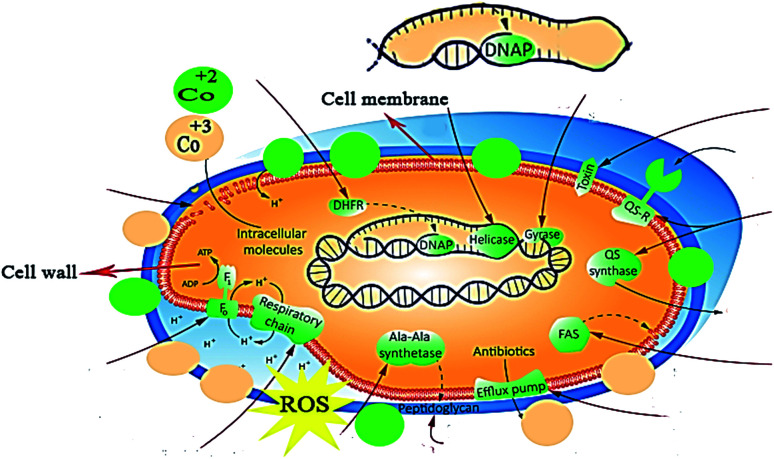
Bactericidal mechanism exhibited by the prepared C_3_N_4_/Fe@Co_3_O_4_ nanocomposites.

Fig. 10 and Table 2 show the *in vitro* antimicrobial action of Co_3_O_4_, Fe@Co_3_O_4_ and C_3_N_4_-doped Fe@Co_3_O_4_ composites *via* agar disk diffusion method against *S. aureus* and *E. coli* obtained from clinically positive Bovine milk. Apart from effective performance of Co_3_O_4_ and Fe@Co_3_O_4_, the synergistic effect of C_3_N_4_-doped Fe@Co_3_O_4_ caused an enhancement in bactericidal activity with increasing amount of C_3_N_4_ in the composite for both (Gram +ve and Gram −ve) bacteria. Significant resistance was shown by synthesized nanocomposites against *S. aureus* with diameter ranging from 3.70–6.00 mm (high dosage) as compared to *E. coli* showing inhibition diameter of 1.40–2.05 mm (high dosage). Control +ve (ciprofloxacin) depicted diameters of 9.15 and 4.25 mm for *S. aureus* and *E. coli*, respectively. Results depicted prominent increment in zone diameter hence indicating enhancement in bactericidal activity upon increasing the concentration of C_3_N_4_ (Fig. 11).

**Table 2:** Antimicrobial activity of prepared samples

Sample	Inhibition zone^a^ (mm)		Inhibition zone^b^ (mm)	
	0.5 mg/50 μL	1.0 mg/50 μL	0.5 mg/50 μL	1.0 mg/50 μL
Co_3_O_4_	2.65	3.70	—	1.40
Fe@Co_3_O_4_	4.10	6.00	—	—
C_3_N_4_ (1%)	2.45	4.55	1.30	1.65
C_3_N_4_ (2%)	3.35	5.30	1.45	1.85
C_3_N_4_ (3%)	3.65	5.65	1.65	2.05
Ciprofloxacin	9.15	9.15	4.25	4.25
DIW	0	0	0	0

a Inhibition zones (mm) measured for *S. aureus*.

b Diameters (mm) observed for *E. coli*.

Bactericidal action of nanostructures is attributed to the formation of reactive oxygen species (ROS) and ionic radical species that interact with cell walls and DNA structure of bacteria. Reactive oxygen species (OH^−^ and H_2_O_2_) are formed in the presence of nano-catalysts under UV-Vis irradiation (electron–hole generation), which induce oxidative stress on the cell walls of bacteria. Another possibility involves the interaction of positively charged Co^2+^ and Co^3+^ with negatively charged cell membrane, resulting in cell death.

Role of nanoparticles as good antibacterial agents is well-documented. Although various mechanisms such as disruption of cell wall synthesis and inhibition of key enzymes of metabolic pathways have been suggested as possible mechanisms governing bactericidal activity of NPs, however exact mechanism still needs to be explored. As key enzymes from cell wall biosynthetic pathway,^77,78^ folate biosynthesis^79,80^ and fatty acid biosynthetic pathway have been reported as attractive target for antibiotic discovery, here we performed molecular docking predictions against enzymes of these selected pathways. Therefore, the binding tendency of C_3_N_4_-doped Fe@Co_3_O_4_ NPs was evaluated against β-lactamase, DHFR and FabI enzymes to analyze their role as inhibitor of these selected targets.

**Fig. 12:**
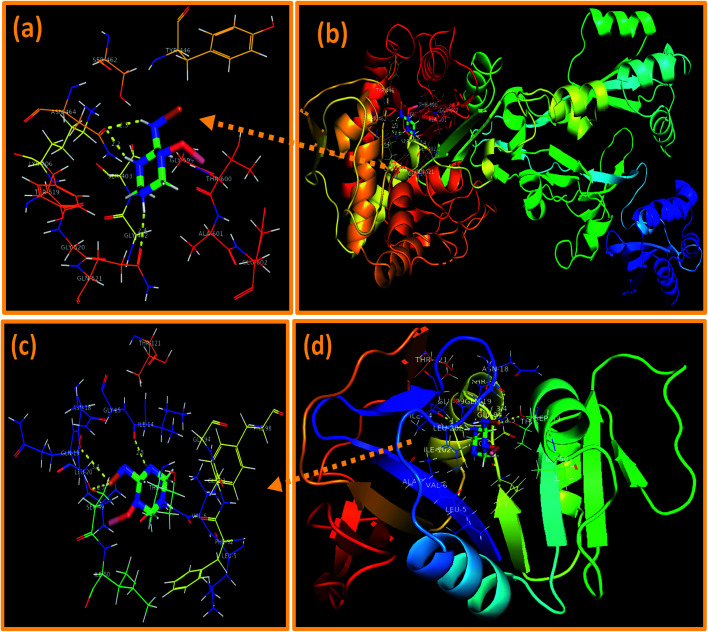
Binding interaction pattern of C_3_N_4_-doped Fe@Co_3_O_4_ NPs inside active site of (a and b). β-Lactamase, (c and d). dihydrofolate reductase (DHFR) from *S. aureus*.

In the case of β-lactamase_*S. aureus*_, the best-docked complex obtained revealed H-bonding interaction of NPs with two amino acid residues *i.e.* Asn464 (2.2 Å and 2.3 Å) and Gln521 (2.4 Å) alongside metal–contact interaction with Thr600 with the overall binding score of −7.833 kcal mol^−1^ as depicted in Fig. 12a and b.

On the other hand, the best-docked conformation of C_3_N_4_-doped Fe@Co_3_O_4_ NPs against active site of DHFR_*S. aureus*_ showed involvement of three amino acid residues of binding pocket in H-bond interaction like Ser49 (2.5 Å), Asn18 (3.4 Å) and Ile14 (2.5 Å) with binding score of −9.418 kcal mol^−1^. Similarly, metal–acceptor bond with Ser49 was also observed as shown in Fig. 12c and d.

**Fig. 13:**
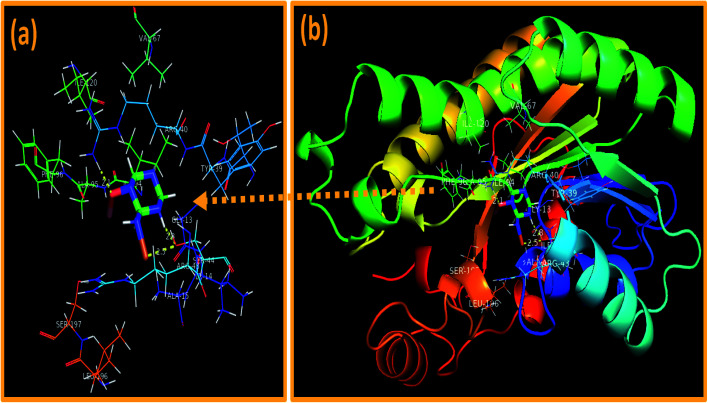
Binding interaction pattern of C_3_N_4_-doped Fe@Co_3_O_4_ NPs inside active site of (a and b). Enoyl-[acyl-carrier-protein] reductase (FabI) from *S. aureus*.

In addition, the docking predictions of C_3_N_4_-doped Fe@Co_3_O_4_ NPs in the case of FabI_*S. aureus*_ showed H-bonding interaction with Ser44 (2.5 Å and 2.8 Å) and Arg40 (2.1 Å) as depicted in Fig. 13a and b with binding score of −5.893 kcal mol^−1^.

*In silico* molecular docking predictions showed good agreement with *in vitro* antibacterial activity of C_3_N_4_-doped Fe@Co_3_O_4_ NPs against *S. aureus* and suggested inhibition of these selected enzyme as a possible mechanism.

## 4 Conclusion

Cost-effective co-precipitation route was adopted to fabricate novel prism-shaped C_3_N_4_-doped Fe@Co_3_O_4_ nanocomposites. Photocatalytic, sonocatalytic and photo-sonocatalytic potentials of synthesized catalysts were evaluated against MBCF dye. An outstanding degradation of >99% was observed for the novel heterojunction framework which followed the type-II transfer mechanism of photogenic excitons. The enhanced performance of the catalyst was attributed to the large surface area, enhanced visible light harvesting capability of Co_3_O_4_ atoms and effective separation of photogenic charge carriers due to the addition of well-matched energy levels of C_3_N_4_. Moreover, the photocatalytic and sonocatalytic activity of prepared catalysts was controlled by tuning the doping concentration of C_3_N_4_ and an optimum doping amount was proposed. In addition to above, the prepared samples also depicted noteworthy bactericidal efficacy against *S. aureus* bacteria where inhibition of β-lactamase, DHFR and FabI enzyme of *S. aureus* was suggested as possible mechanism behind their bactericidal potential. Overall, this study offers new insights into the use of cobalt-based heterojunction photocatalysts for various photocatalytic, sonocatalytic and antibacterial applications.

## Funding

Authors are thankful to higher education commission (HEC), Pakistan through project 21-1669/SRGP/R&D/HEC/2017. Support provided by the Core Research Facilities at King Fahd University of Petroleum & Minerals, Dhahran, Saudi Arabia is highly appreciated.

## Availability of data and materials

All data are fully available without restriction.

## Author contributions

SOAA performed the whole experiments and wrote the manuscript. MI and M. Imran provided the novel idea to carry out the experiment. AH performed antimicrobial and participated in the data analysis of the results and discussion portion. S. Naz and J. Haider performed molecular docking study. A Shahzadi worked on schematic diagram and reviewed the manuscript. AUH carried out the FESEM and HRTEM analysis. All authors read and approved the final manuscript.

## Conflicts of interest

Authors confirm no conflict of interest.

## Supplementary Material

## References

[cit1] Zhao H., Xia Q., Xing H., Chen D., Wang H. (2017). Construction of Pillared-Layer MOF as Efficient Visible-Light Photocatalysts for Aqueous Cr(VI) Reduction and Dye Degradation. ACS Sustain. Chem. Eng..

[cit2] Dias E. M., Petit C. (2015). Towards the use of metal-organic frameworks for water reuse: A review of the recent advances in the field of organic pollutants removal and degradation and the next steps in the field. J. Mater. Chem. A..

[cit3] Wang C. C., Li J. R., Lv X. L., Zhang Y. Q., Guo G. (2014). Photocatalytic organic pollutants degradation in metal-organic frameworks. Energy Environ. Sci..

[cit4] Wang S., Guan B. Y., Lou X. W. D. (2018). Construction of ZnIn2S4-In2O3 Hierarchical Tubular Heterostructures for Efficient CO2 Photoreduction. J. Am. Chem. Soc..

[cit5] Ali I., Asim M., Khan T. A. (2012). Low cost adsorbents for the removal of organic pollutants from wastewater. J. Environ. Manage..

[cit6] Yang M. (2011). A current global view of environmental and occupational cancers. J. Environ. Sci. Heal. - Part C Environ. Carcinog. Ecotoxicol. Rev..

[cit7] Temesgen T., Bui T. T., Han M., il Kim T., Park H. (2017). Micro and nanobubble technologies as a new horizon for water-treatment techniques: A review. Adv. Colloid Interface Sci..

[cit8] Ali I. (2012). New generation adsorbents for water treatment. Chem. Rev..

[cit9] Lin S. H., Chen M. L. (1997). Treatment of textile wastewater by-chemical methods for reuse. Water Res..

[cit10] Goldberg S. (2009). Influence of soil solution salinity on molybdenum adsorption by soils. Soil Sci..

[cit11] Ben Aim R. , Liu M. G. and Vigneswaran S., Recent development of membrane processes for water and waste water treatment, in Water Sci. Technol., IWA Publishing, 1993, pp. 141–149. 10.2166/wst.1993.0221

[cit12] Särkkä H., Bhatnagar A., Sillanpää M. (2015). Recent developments of electro-oxidation in water treatment - A review. J. Electroanal. Chem..

[cit13] Chong M. N., Jin B., Chow C. W. K., Saint C. (2010). Recent developments in photocatalytic water treatment technology: A review. Water Res..

[cit14] Litter M. I. (1999). Heterogeneous photocatalysis: Transition metal ions in photocatalytic systems. Appl. Catal., B.

[cit15] Li G. Z., Yu M., Wang Z. L., Lin J., Wang R. S., Fang J. (2006). Sol-gel fabrication and photoluminescence properties of SiO 2@Gd 2O 3:Eu 3+ core-shell particles. J. Nanosci. Nanotechnol..

[cit16] Lü J., Lin J. X., Zhao X. L., Cao R. (2012). Photochromic hybrid materials of cucurbituril and polyoxometalates as photocatalysts under visible light. Chem. Commun..

[cit17] Thompson T. L., Yates J. T. (2006). Surface science studies of the photoactivation of TIO2 - New photochemical processes. Chem. Rev..

[cit18] Ayoub K., van Hullebusch E. D., Cassir M., Bermond A. (2010). Application of advanced oxidation processes for TNT removal: A review. J. Hazard. Mater..

[cit19] Du J. J., Yuan Y. P., Sun J. X., Peng F. M., Jiang X., Qiu L. G., Xie A. J., Shen Y. H., Zhu J. F. (2011). New photocatalysts based on MIL-53 metal-organic frameworks for the decolorization of methylene blue dye. J. Hazard. Mater..

[cit20] Wang H., Zhang L., Chen Z., Hu J., Li S., Wang Z., Liu J., Wang X. (2014). Semiconductor heterojunction photocatalysts: Design, construction, and photocatalytic performances. Chem. Soc. Rev..

[cit21] Kuriki R., Yamamoto M., Higuchi K., Yamamoto Y., Akatsuka M., Lu D., Yagi S., Yoshida T., Ishitani O., Maeda K. (2017). Robust Binding between Carbon Nitride Nanosheets and a Binuclear Ruthenium(II) Complex Enabling Durable, Selective CO 2 Reduction under Visible Light in Aqueous Solution. Angew. Chem..

[cit22] Kuriki R., Matsunaga H., Nakashima T., Wada K., Yamakata A., Ishitani O., Maeda K. (2016). Nature-Inspired, Highly Durable CO2 Reduction System Consisting of a Binuclear Ruthenium(II) Complex and an Organic Semiconductor Using Visible Light. J. Am. Chem. Soc..

[cit23] Wang K., Huang Z., Jin X., Zhang D., Wang J. (2021). MOF–derived hollow porous ZnFe2O4/AgCl/Ag/C nanotubes with magnetic–dielectric synergy as high–performance photocatalysts for hydrogen evolution reaction. Chem. Eng. J..

[cit24] Wang K., Zhan S., Sun H., Zhang D., Wang J. (2020). Hollow porous core–shell ZnFe2O4/AgCl nanocubes coated with EDTA and Ag nanoparticles for enhanced photocatalytic performances of visible–light–driven. Chem. Eng. J..

[cit25] Jabeen U., Shah S. M., Khan S. U. (2017). Photo catalytic degradation of Alizarin red S using ZnS and cadmium doped ZnS nanoparticles under unfiltered sunlight. Surf. Interfaces.

[cit26] Wang X., Maeda K., Thomas A., Takanabe K., Xin G., Carlsson J. M., Domen K., Antonietti M. (2009). A metal-free polymeric photocatalyst for hydrogen production from water under visible light. Nat. Mater..

[cit27] Martin D. J., Qiu K., Shevlin S. A., Handoko A. D., Chen X., Guo Z., Tang J. (2014). Highly Efficient Photocatalytic H 2 Evolution from Water using Visible Light and Structure-Controlled Graphitic Carbon Nitride. Angew. Chem., Int. Ed..

[cit28] Mao Z., Chen J., Yang Y., Wang D., Bie L., Fahlman B. D. (2017). Novel g-C3N4/CoO Nanocomposites with Significantly Enhanced Visible-Light Photocatalytic Activity for H2 Evolution. ACS Appl. Mater. Interfaces.

[cit29] Wang X., Maeda K., Chen X., Takanabe K., Domen K., Hou Y., Fu X., Antonietti M. (2009). Polymer semiconductors for artificial photosynthesis: Hydrogen evolution by mesoporous graphitic carbon nitride with visible light. J. Am. Chem. Soc..

[cit30] Zhang Y., Liu J., Wu G., Chen W. (2012). Porous graphitic carbon nitride synthesized via direct polymerization of urea for efficient sunlight-driven photocatalytic hydrogen production. Nanoscale.

[cit31] Li X. H., Wang X., Antonietti M. (2012). Mesoporous g-C 3N 4 nanorods as multifunctional supports of ultrafine metal nanoparticles: Hydrogen generation from water and reduction of nitrophenol with tandem catalysis in one step. Chem. Sci..

[cit32] McDaniel H., Heil P. E., Tsai C. L., Kim K., Shim M. (2011). Integration of type II nanorod heterostructures into photovoltaics. ACS Nano.

[cit33] Shim M., McDaniel H., Oh N. (2011). Prospects for strained type-II nanorod heterostructures. J. Phys. Chem. Lett..

[cit34] Hou Y., Zhu Y., Xu Y., Wang X. (2014). Photocatalytic hydrogen production over carbon nitride loaded with WS2 as cocatalyst under visible light. Appl. Catal., B.

[cit35] Yan J., Wu H., Chen H., Zhang Y., Zhang F., Liu S. F. (2016). Fabrication of TiO2/C3N4 heterostructure for enhanced photocatalytic Z-scheme overall water splitting. Appl. Catal., B.

[cit36] Kumar S., Baruah A., Tonda S., Kumar B., Shanker V., Sreedhar B. (2014). Cost-effective and eco-friendly synthesis of novel and stable N-doped ZnO/g-C3N4 core-shell nanoplates with excellent visible-light responsive photocatalysis. Nanoscale.

[cit37] Liao L., Zhang Q., Su Z., Zhao Z., Wang Y., Li Y., Lu X., Wei D., Feng G., Yu Q., Cai X., Zhao J., Ren Z., Fang H., Robles-Hernandez F., Baldelli S., Bao J. (2014). Efficient solar water-splitting using a nanocrystalline CoO photocatalyst. Nat. Nanotechnol..

[cit38] Liu Y., Ding S., Shi Y., Liu X., Wu Z., Jiang Q., Zhou T., Liu N., Hu J. (2018). Construction of CdS/CoOx core-shell nanorods for efficient photocatalytic H2 evolution. Appl. Catal., B.

[cit39] Guo F., Shi W., Zhu C., Li H., Kang Z. (2018). CoO and g-C3N4 complement each other for highly efficient overall water splitting under visible light. Appl. Catal., B.

[cit40] Jin J., Fu X., Liu Q., Zhang J. (2013). A highly active and stable electrocatalyst for the oxygen reduction reaction based on a graphene-supported g-C3N4@cobalt oxide core-shell hybrid in alkaline solution. J. Mater. Chem. A..

[cit41] Hai C., Li S., Zhou Y., Zeng J., Ren X., Li X. (2017). Roles of ethylene glycol solvent and polymers in preparing uniformly distributed MgO nanoparticles. J. Asian Ceram. Soc..

[cit42] Lim D., Strynadka N. C. J. (2002). Structural basis for the β-lactam resistance of PBP2a from methicillin-resistant Staphylococcus aureus. Nat. Struct. Biol..

[cit43] Heaslet H., Harris M., Fahnoe K., Sarver R., Putz H., Chang J., Subramanyam C., Barreiro G., Miller J. R. (2009). Structural comparison of chromosomal and exogenous dihydrofolate reductase from Staphylococcus aureus in complex with the potent inhibitor trimethoprim. Proteins Struct. Funct. Bioinforma..

[cit44] Fage C. D., Lathouwers T., Vanmeert M., Gao L., Vrancken K., Lammens E., Weir A. N. M., Degroote R., Cuppens H., Kosol S., Simpson T. J., Crump M. P., Willis C. L., Herdewijn P., Lescrinier E., Lavigne R., Anné J., Masschelein J. (2020). The Kalimantacin Polyketide Antibiotics Inhibit Fatty Acid Biosynthesis in Staphylococcus aureus by Targeting the Enoyl-Acyl Carrier Protein Binding Site of FabI. Angew. Chem..

[cit45] Vilar S., Cozza G., Moro S. (2008). Medicinal Chemistry and the Molecular
Operating Environment (MOE): Application of QSAR and Molecular Docking to Drug Discovery. Curr. Top. Med. Chem..

[cit46] Ikram M., Hassan J., Raza A., Haider A., Naz S., Ul-Hamid A., Haider J., Shahzadi I., Qamar U., Ali S. (2020). Photocatalytic and bactericidal properties and molecular docking analysis of TiO2nanoparticles conjugated with Zr for environmental remediation. RSC Adv..

[cit47] Ikram M., Abbasi S., Haider A., Naz S., Ul-Hamid A., Imran M., Haider J., Ghaffar A. (2020). Bimetallic Ag/Cu incorporated into chemically exfoliated MoS2 nanosheets to enhance its antibacterial potential: In silico molecular docking studies. Nanotechnology.

[cit48] Yuan Y. F., Xia X. H., Wu J. B., Gui J. S., Chen Y. B., Guo S. Y. (2010). Electrochromism in mesoporous nanowall cobalt oxide thin films prepared via lyotropic liquid crystal media with electrodeposition. J. Memb. Sci..

[cit49] Zhang J., Wang X., Qin D., Xue Z., Lu X. (2014). Fabrication of iron-doped cobalt oxide nanocomposite films by electrodeposition and application as electrocatalyst for oxygen reduction reaction. Appl. Surf. Sci..

[cit50] Xia X. H., Tu J. P., Xiang J. Y., Huang X. H., Wang X. L., Zhao X. B. (2010). Hierarchical porous cobalt oxide array films prepared by electrodeposition through polystyrene sphere template and their applications for lithium ion batteries. J. Power Sources.

[cit51] Shim H. S., Shinde V. R., Kim H. J., Sung Y. E., Kim W. B. (2008). Porous cobalt oxide thin films from low temperature solution phase synthesis for electrochromic electrode. Thin Solid Films.

[cit52] Babar S., Gavade N., Shinde H., Gore A., Mahajan P., Lee K. H., Bhuse V., Garadkar K. (2019). An innovative transformation of waste toner powder into magnetic g-C3N4-Fe2O3 photocatalyst: Sustainable e-waste management. J. Environ. Chem. Eng..

[cit53] Transactions C. S., Manigandan R., Giribabu K., Suresh R., Vijayalakshmi L., Stephen A., Narayanan V., Campus G., Campus G. (2013). Cobalt Oxide Nanoparticles: Characterization and its Electrocatalytic Activity towards Nitrobenzene. Chem. Sci. Trans..

[cit54] Durai D., Prabaharan M., Sadaiyandi K., Mahendran M., Sagadevan S. (2017). Precipitation method and characterization of cobalt oxide nanoparticles. Appl. Phys. A.

[cit55] Mu X., Pan Y., Ma C., Zhan J., Song L. (2018). Novel Co3O4/covalent organic frameworks nanohybrids for conferring enhanced flame retardancy, smoke and CO suppression and thermal stability to polypropylene. Mater. Chem. Phys..

[cit56] Xie X., Shang P., Liu Z., Lv Y., Li Y., Shen W. (2010). Synthesis of nanorod-shaped cobalt hydroxycarbonate and oxide with the mediation of ethylene glycol. J. Phys. Chem. C.

[cit57] Klissurski D. G., Uzunova E. L. (1991). Synthesis of Nickel Cobaltite Spinel from Coprecipitated Nickel-Cobalt Hydroxide Carbonate. Chem. Mater..

[cit58] Xu R., Zeng H. C. (2003). Dimensional Control of Cobalt-hydroxide-carbonate Nanorods and Their Thermal Conversion to One-dimensional Arrays of Co3O4 Nanoparticles. J. Phys. Chem. B.

[cit59] Barreca D., Massignan C., Daolio S., Fabrizio M., Piccirillo C., Armelao L., Tondello E. (2001). Composition and microstructure of cobalt oxide thin films obtained from a novel cobalt(II) precursor by chemical vapor deposition. Chem. Mater..

[cit60] Deori K., Ujjain S. K., Sharma R. K., Deka S. (2013). Morphology controlled synthesis of nanoporous Co3O4 nanostructures and their charge storage characteristics in supercapacitors. ACS Appl. Mater. Interfaces.

[cit61] Kim K. J., Park Y. R. (2003). Optical investigation of charge-transfer transitions in spinel Co3O4. Solid State Commun..

[cit62] Chen X., Chen J., Qiao X., Wang D., Cai X. (2008). Performance of nano-Co3O4/peroxymonosulfate system: Kinetics and mechanism study using Acid Orange 7 as a model compound. Appl. Catal., B.

[cit63] Patil P. S., Kadam L. D., Lokhande C. D. (1996). Preparation and characterization of spray pyrolysed cobalt oxide thin films. Thin Solid Films.

[cit64] Shockley W., Read W. T. (1952). Statistics of the recombinations of holes and electrons. Phys. Rev..

[cit65] Xing L. L., Chen Z. H., Xue X. Y. (2014). Controllable synthesis Co3O4 nanorods and nanobelts and their excellent lithium storage performance. Solid State Sci..

[cit66] Du X., Skachko I., Barker A., Andrei E. Y. (2008). Approaching ballistic transport in suspended graphene. Nat. Nanotechnol..

[cit67] Tuinstra F., Koenig J. (1970). Raman spectrum of graphite. J. Chem. Phys..

[cit68] Dutta P., Seehra M. S., Thota S., Kumar J. (2008). A comparative study of the magnetic properties of bulk and nanocrystalline Co3O4. J. Phys. Condens. Matter..

[cit69] Farhadi S., Safabakhsh J., Zaringhadam P. (2013). Synthesis, characterization, and investigation of optical and magnetic properties of cobalt oxide (Co3O4) nanoparticles. J. Nanostructure Chem..

[cit70] Salavati-Niasari M., Mir N., Davar F. (2009). Synthesis and characterization of Co3O4 nanorods by thermal decomposition of cobalt oxalate. J. Phys. Chem. Solids.

[cit71] Stella C., Soundararajan N., Ramachandran K. (2015). Structural, optical, and magnetic properties of Mn and Fe-doped Co3O4 nanoparticles. AIP Adv.

[cit72] Kite S. V., Sathe D. J., Kadam A. N., Chavan S. S., Garadkar K. M. (2020). Highly efficient photodegradation of 4-nitrophenol over the nano-TiO2 obtained from chemical bath deposition technique. Res. Chem. Intermed..

[cit73] Khataee A., Sheydaei M., Hassani A., Taseidifar M., Karaca S. (2015). Sonocatalytic removal of an organic dye using TiO2/Montmorillonite nanocomposite. Ultrason. Sonochem..

[cit74] Agarwal A., Ng W. J., Liu Y. (2011). Principle and applications of microbubble and nanobubble technology for water treatment. Chemosphere.

[cit75] Wang J., Jiang Z., Zhang Z., Xie Y., Lv Y., Li J., Deng Y., Zhang X. (2009). Study on inorganic oxidants assisted sonocatalytic degradation of Acid Red B in presence of nano-sized ZnO powder. Sep. Purif. Technol..

[cit76] Goh X. , Synthesis of TiO2/G-C3N4 Composite for Sonocatalytic Degradation of Organic Dye 2018, http://eprints.utar.edu.my/3115/1/CL-2018-1407419-1.pdf, accessed June 19, 2021

[cit77] Page M. G. P. , Beta-lactam antibiotics, in Antibiot. Discov. Dev., Springer US, 2012, pp. 79–117. 10.1007/978-1-4614-1400-1_3

[cit78] Paterson D. L., Ko W. C., Von Gottberg A., Casellas J. M., Mulazimoglu L., Klugman K. P., Bonomo R. A., Rice L. B., McCormack J. G., Yu V. L. (2001). Outcome of cephalosporin treatment for serious infections due to apparently susceptible organisms producing extended-spectrum $β$-lactamases: Implications for the clinical microbiology laboratory. J. Clin. Microbiol..

[cit79] Dale G. E., Broger C., D'Arcy A., Hartman P. G., DeHoogt R., Jolidon S., Kompis I., Labhardt A. M., Langen H., Locher H., Page M. G. P., Stüber D., Then R. L., Wipf B., Oefner C. (1997). A single amino acid substitution in Staphylococcus aureus dihydrofolate reductase determines trimethoprim resistance. J. Mol. Biol..

[cit80] Hawser S., Lociuro S., Islam K. (2006). Dihydrofolate reductase inhibitors as antibacterial agents. Biochem. Pharmacol..

